# Different Machine Learning and Deep Learning Methods for the Classification of Colorectal Cancer Lymph Node Metastasis Images

**DOI:** 10.3389/fbioe.2020.620257

**Published:** 2021-01-14

**Authors:** Jin Li, Peng Wang, Yang Zhou, Hong Liang, Kuan Luan

**Affiliations:** ^1^College of Intelligent System Science and Engineering, Harbin Engineering University, Harbin, China; ^2^Department of Radiology, Harbin Medical University Cancer Hospital, Harbin, China

**Keywords:** colorectal cancer, lymph node, classification, transfer learning, deep learning

## Abstract

The classification of colorectal cancer (CRC) lymph node metastasis (LNM) is a vital clinical issue related to recurrence and design of treatment plans. However, it remains unclear which method is effective in automatically classifying CRC LNM. Hence, this study compared the performance of existing classification methods, i.e., machine learning, deep learning, and deep transfer learning, to identify the most effective method. A total of 3,364 samples (1,646 positive and 1,718 negative) from Harbin Medical University Cancer Hospital were collected. All patches were manually segmented by experienced radiologists, and the image size was based on the lesion to be intercepted. Two classes of global features and one class of local features were extracted from the patches. These features were used in eight machine learning algorithms, while the other models used raw data. Experiment results showed that deep transfer learning was the most effective method with an accuracy of 0.7583 and an area under the curve of 0.7941. Furthermore, to improve the interpretability of the results from the deep learning and deep transfer learning models, the classification heat-map features were used, which displayed the region of feature extraction by superposing with raw data. The research findings are expected to promote the use of effective methods in CRC LNM detection and hence facilitate the design of proper treatment plans.

## Introduction

Colorectal cancer (CRC) has a higher recurrence rate than all other cancers (Bray et al., 2018). CRC lymph node metastasis (LNM) is the root cause of CRC recurrence (Ding et al., 2019; Yang and Liu, 2020). CRC patients with LNM have a 5-year survival rate ranging from 50 to 68%, but those without LNM have a higher rate up to 95% (Ishihara et al., 2017; Zhou et al., 2017). Treatment of CRC is also influenced by the presence of LNM. The conventional treatment plan involves endoscopic resection, and surgical resection accompanied by LN dissection is necessary for patients with LNM (Nasu et al., 2013). Hence, it is important to determine the presence of CRC LNM, and to this end, an automatic classification method for CRC LNM should be explored to give a second objective opinion and then assist the radiologist in providing a correct report.

As computer technologies advance in these years, medical imaging classification methods have seen wider adoption. Many new methods based on machine learning (Al-Absi et al., 2012), deep learning (Sun et al., 2016), or deep transfer learning (Pratap and Kokil, 2019), have gradually been applied to medical imaging classification. These methods can provide additional preoperative information to aid radiologists in making proper treatment plans (Carneiro et al., 2017; Lu et al., 2017).

There are many types of machine learning algorithms that can be applied to medical imaging classification, such as support vector machine (SVM) (Burges, 1998), decision trees (DT) (Quinlan, 1986), and naïve Bayes (NB) (Friedman et al., 1997). Vibha et al. (2006) used DT to classify mammograms and obtained a classification accuracy of almost 90%. Inthajak et al. (2011) presented a k-nearest neighbor (KNN) algorithm to categorize medical images. Ahmad et al. (2016) used NB classification to categorize each chest X-ray image to either normal with infection or with fluid in capture features. García-Floriano et al. (2019) proposed a machine learning model based on SVM. This model could classify age-related macular degeneration in fundus images and achieved a higher classification accuracy than many well-regarded state-of-the-art methods. Luo et al. (2018) used SVM to classify human stomach cancer in optical coherence tomography images and obtained a higher classification accuracy than human detection. Although these methods outperformed human radiologists in terms of classification accuracy, they are subject to problems in feature extraction. The features that are often manually extracted fall short of objectivity and will affect the algorithms' performance and hence the classification accuracy. Thus, a method that could learn underlying data features is needed.

Deep learning (Lecun et al., 2015) has achieved stunning success in image classification in the ImageNet Large Scale Visual Recognition Challenge (Krizhevsky et al., 2017) in 2012. There are several landmark studies (Ma et al., 2017; Golatkar et al., 2018) that have promoted the development of deep learning algorithms. Compared to machine learning, deep learning has a vital advantage, i.e., the ability to automatically learn the potential features of data by utilizing the convolution neural network (CNN). A CNN could automatically learn potential features from raw data layer by layer with little or no hands-on intervention. Now, CNNs have already become a study focus, especially in medical imaging (Litjens et al., 2017; Shen et al., 2017). For instance, Song et al. (2017) used a CNN for lung nodule classification on computed tomography (CT). Liu and An (2017) built a classification model for detection of prostate cancer based on deep learning. Despite the good performance of deep learning methods in image classification, however, there are two problems that undermine their wider adoption – the need for massive number of data and high-performance computing devices, like graphic processing units (GPUs). As deep learning needs a large number of data to train and fit the CNN parameters, GPUs are preferred than other equipment to process the images faster, but it incurs a high training cost. Thus, reducing the training cost is the key to solving the problem.

Transfer learning, introduced by Pan and Yang (2010), uses the existing knowledge learned from one environment to solve similar problems in different environments. Pan and Yang (2010) summarized the classification process as well as the pros and cons of transfer learning methods. Transfer learning methods have a lower training cost than their deep learning counterparts as the former does not require as many data as the latter needs for training. In transfer learning, a model pretrained on another large dataset, such as ImageNet, is employed to complete a task through fine-tuning^1^ or other methods (Long et al., 2013, 2017; Tzeng et al., 2014), so it has a lower cost than deep learning. Because of these advantages, transfer learning is widely used in medical imaging. Vesal et al. (2018) used two pre-trained models to classify breast cancer histology images and obtained an accuracy of 97.50 and 91.25%. da Nobrega et al. (2018) used a deep transfer learning model (Tan et al., 2018) to classify lung nodules in CT lung images. Dornaika et al. (2019) used transfer learning to estimate age through facial images, and the experiments were carried out on three public databases.

In previous studies, comparisons were made to find the most effective method for specific diseases. Wang L. et al. (2017) used four methods with eight classification schemes to classify ophthalmic images and found that local binary pattern with SVM and wavelet transformation with SVM had the best classification performance, with an accuracy of 87%. Wang H. et al. (2017) used five methods, including random forest, SVM, adaptive boosting, back-propagation artificial neural network (ANN), and CNN to classify mediastinal LNM of non-small cell lung cancer. Lee et al. (2019) used eight deep learning algorithms to differentiate benign and malignant tumors from cervical metastatic LN of thyroid cancer based on preoperative CT images, trying to identify the most suitable model. However, to the authors' knowledge, there are few methods used for CRC LNM classification based on machine learning, deep learning, or deep transfer learning. Furthermore, no previous studies have compared these three methods. Therefore, which method is the most effective one for CRC LNM classification is unclear.

According to the literature, the use of machine learning, deep learning, or transfer learning in medical imaging is rapidly developing and evolving. Using computational approaches to provide additional preoperative information can help doctors design proper treatment plans (Carneiro et al., 2017; Lu et al., 2017). Several papers have been published on CRC (Simjanoska et al., 2013; Ciompi et al., 2017; Nakaya et al., 2017; Bychkov et al., 2018), but few studies have explored the performance of machine learning, deep learning, and transfer learning in CRC LNM classification, and which method has the best performance is yet to be determined, which motivated us to conduct this research. To identify the most effective method for CRC LNM classification, the following approaches were taken in this study.

First, eight machine learning, two deep learning, and one transfer learning classification methods were used to classify CRC LNM images.

Next, the classification results were compared to evaluate the performance of different methods and identify the one with the best classification performance.

Finally, a classification heat-map was used to improve the interpretability of the classification method for CRC LNM.

## Materials and Methods

### Data

**Figure 1:**
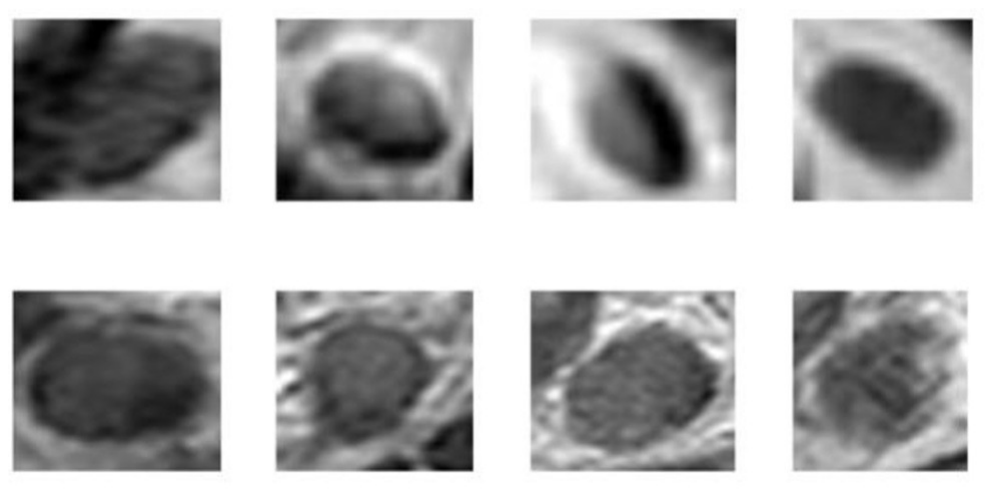
Sample CRC LN images (top row, negative; bottom row, positive).

Data used in the present study were collected from Harbin Medical University Cancer Hospital. There were 3,364 samples in the dataset, among which 1,646 were positive and 1,718 were negative. The standard of all samples was an LN of a diameter >3 mm. All patients underwent 3.0T magnetic resonance imaging (MRI) before surgery using Philips Achieva, with a 16-channel torso array coil. MRI sagittal T2WI scan sequence was performed with the following parameters: TR/TE 3000/100 ms, the number of signal frequency (NSA) 2, and the layer thickness 4.0 mm, and layer spacing 0.4 mm. The position of the rectal lesions was determined by the sagittal position, which was perpendicular to the intestinal canal lesions, with a transverse T2WI scan: TR/TE 3,824/110 ms, NSA 0–3, layer thickness 3.5 mm, and interval 0.2 mm. According to the sagittal lesion position, patients with parallel pathological changes received coronal T2WI scans: TR/TE 3,824/110 ms, NSA 3, and layer spacing 0.2 mm. Then, the objective LN in the sagittal, transverse, and coronal images were located. All images used in this study were marked as CRC LNs and classified as negative or positive by experienced radiologists. All patches were manually segmented by experienced radiologists, and the image size was based on the lesion to be intercepted. Figure 1 presents the CRC LN.

### Methods

In this study, intensity features were extracted by a gray-level histogram (GLH) (Otsu, 1979), and the textural features were extracted by the gray-level co-occurrence matrix (GLCM) (Haralick et al., 1973). These two features are global features. Furthermore, the scale-invariant feature transform (SIFT) (Lowe, 1999, 2004) operator was used for local features. These methods were implemented in Python.

Eight classical supervised machine learning methods, including AdaBoost (AB) (Freund and Schapire, 1997), DT (Quinlan, 1986), KNN (Cover and Hart, 1967), logistic regression (LR) (Cucchiara, 2012), NB (Friedman et al., 1997), SVM (Burges, 1998), stochastic gradient descent (SGD) (Ratnayake et al., 2014), and multilayer perceptron (MLP), two deep learning models [LeNet (Lecun et al., 1998) and AlexNet (Krizhevsky et al., 2017)], and one transfer learning model (AlexNet pre-trained model) were studied for CRC LNM classification. The results of these methods were compared.

A detailed introduction to classical machine learning methods was presented in Marsland (2009). Classical machine learning methods were implemented using Python and the sckit-learn package. The optimal parameters of each method were searched via grid search in the parameter space and determined based on the four folds of training samples (Wang H. et al., 2017). AB used 100 decision stumps as weak learners, the learning rate was 0.1, and the maximum split number was equal to 1. DT used sckit-learn package default parameters. The KNN used 10 points of nearest neighbors. LR was a linear classification model, for which the parameters of the norm were l2, the optimization algorithm was selected as linear, and iteration was 100. The NB classifier selected Gaussian NB from the sckit-learn package. SVM used a radial basis function as the kernel function, the kernel coefficient was 1e-3, and the penalty parameter was 1.0. SGD employed hinge as the loss function and l2 as a penalty, and the iteration was 100. MLP, a form of ANN, was trained with a back-propagation algorithm (Rumelhart et al., 1985). In this method, two hidden layers with 32 and 16 neurons for the first and second layers were used. Epochs were 200, and the learning rate was 1e-3.

Deep learning methods were implemented by Python and Keras library for Python. LeNet and AlexNet's structures were based on Lecun et al. (1998) and Krizhevsky et al. (2017). A relu activation function was used in the convolution layer, and a sigmoid function in the full connection layer. The optimization function was SGD (Bottou, 2010). The loss function was binary_crossentropy. The learning rate was 1e-3, and epochs were 200. To avoid overfitting, L2 normalization (Van Laarhoven, 2017) and dropout regularization (Srivastava et al., 2014) were utilized. The methods were running on GPUs (NVIDIA Company GTX 1080ti).

Transfer learning and deep learning share something in common in parameter settings, but are different in training: deep learning starts from scratch, whereas transfer learning uses a pre-trained model. Because the pre-trained model was not trained by ImageNet, it did not need to initialize parameters. As stated in Yosinski et al. (2014), the first three layers were frozen because the layers that extracted the features were general. The other parameters were fine-tuned. In the implementation process, the structure must follow AlexNet then load AlexNet model weight. The fully connected layer activation function was changed to sigmoid. The optimization function was SGD. The loss function was binary_crossentropy, and the learning rate was 1e-4, and epochs were 200. In this study, methods used to avoid overfitting were L2 normalization and dropout regularization. The dropout was set to 0.5. Finally, transfer learning retrained on GPU was like deep learning.

The explanation of the internal relationship between the input data and label prediction has always been a vital issue (Lipton, 2018) in the CNN-based classification tasks. In this study, a classification heat-map was used to improve the interpretability of the model (Lee et al., 2019). This experiment contained three steps. First, the model was used to display the last convolutional layer feature-map. Then, the feature-map was converted into a heat-map. Last, raw data and heat-map were superimposed into a new image.

## Results

### Classification Performance

**Figure 2:**
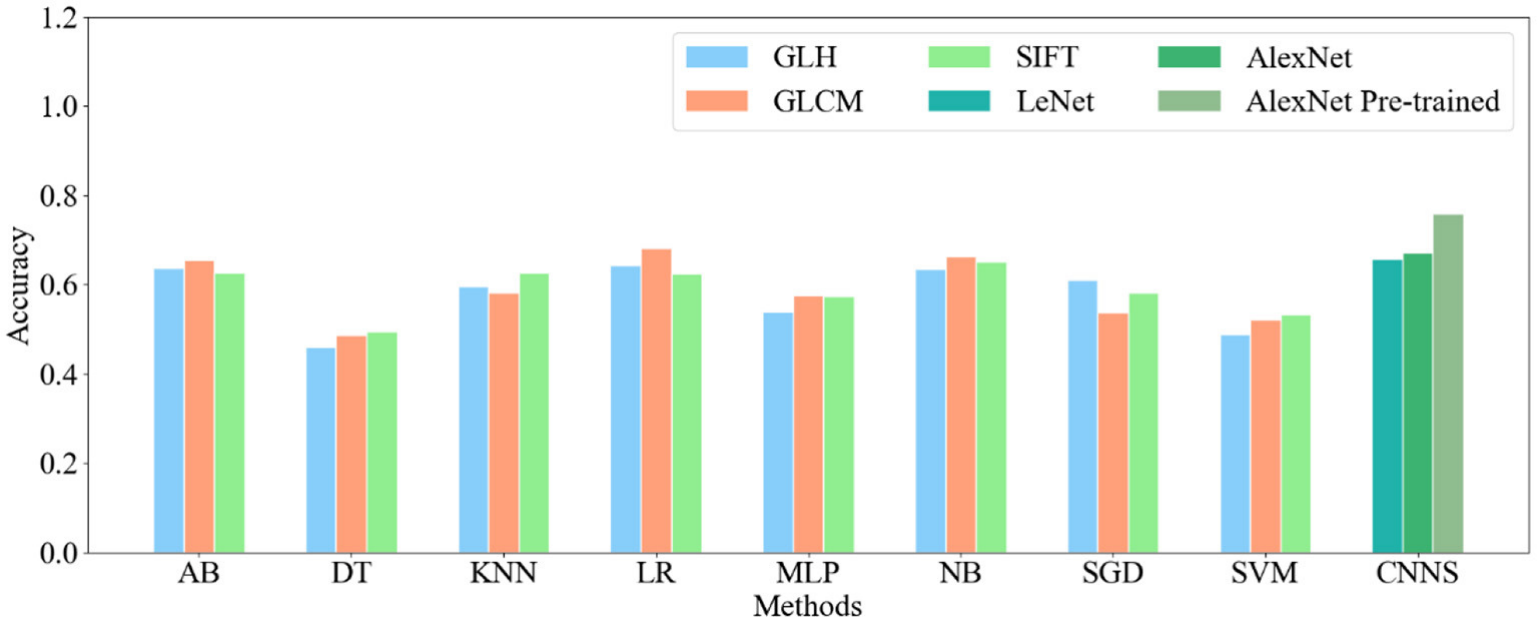
Accuracy of all classification methods.

**Figure 3:**
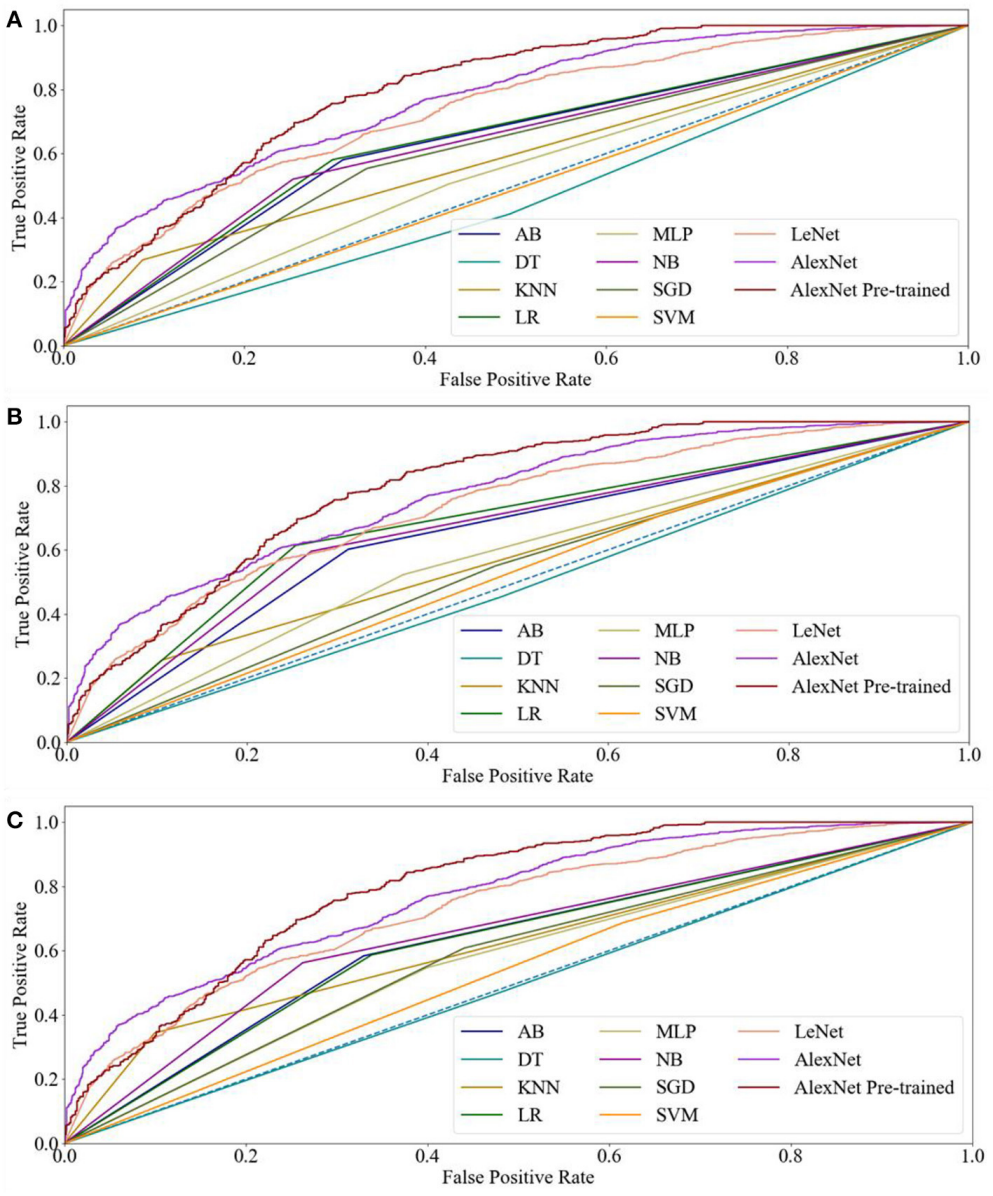
Receiver operating characteristic curves of all methods for CRC LNM: **(A)** ML with GLH and CNN, **(B)** ML with GLCM and CNN, and **(C)** ML with SIFT and CNN.

In this study, three kinds of features were employed in eight machine learning methods, respectively. Table 1 displays the performance indicators of all methods for CRC LNM classification, including accuracy (ACC), area under the curve (AUC), sensitivity, specificity, positive predictive value (PPV), and negative predictive value (NPV). Based on the ACC and AUC values, the optimal features set of each machine learning method for CRC LNM classification was defined. As shown in Figures 2, 3, LR achieved the best performance using the GLH and GLCM features sets. NB obtained the highest ACC and AUC values for the SIFT features set. Deep learning and transfer learning had significantly better performance than machine learning methods in CRC LNM classification. Although deep learning and transfer learning did not use global and local features sets, the ACC and AUC values of these two methods were still better than machine learning methods (Table 1). Hence, transfer learning was identified as the best method for CRC LNM classification in this study.

**Table 1:** Performance results of all methods for CRC LNM.

	**ACC**	**AUC**	**Sensitivity**	**Specificity**	**PPV**	**NPV**
AB+GLH	0.6369	0.6357	0.6753	0.6431	0.6910	0.5805
AB+GLCM	0.6458	0.6449	0.6431	0.6491	0.6880	0.6018
AB+SIFT	0.6280	0.6270	0.6267	0.6295	0.6706	0.5836
DT+GLH	0.4598	0.4588	0.4728	0.4441	0.5073	0.4103
DT+GLCM	0.4866	0.4859	0.4972	0.4745	0.5190	0.4529
DT+SIFT	0.4955	0.4950	0.5057	0.4844	0.5190	0.4711
KNN+GLH	0.5967	0.5900	0.5650	0.7458	0.7125	0.2675
KNN+GLCM	0.5818	0.5752	0.5562	0.7	0.7051	0.2553
KNN+SIFT	0.6280	0.6222	0.5886	0.7687	0.7009	0.3435
LR+GLH	0.6429	0.6416	0.6359	0.6519	0.7026	0.5805
LR+GLCM	0.6815	0.6802	0.6684	0.6990	0.7464	0.6140
LR+SIFT	0.6250	0.6242	0.6253	0.6246	0.6618	0.5866
MLP+GLH	0.5402	0.5395	0.5472	0.5321	0.5743	0.5046
MLP+GLCM	0.5759	0.5748	0.5780	0.5733	0.6268	0.5228
MLP+SIFT	0.5744	0.5738	0.5803	0.5678	0.6006	0.5471
NB+GLH	0.6354	0.6331	0.6184	0.6628	0.7464	0.5198
NB+GLCM	0.6637	0.6623	0.6527	0.6782	0.7289	0.5957
NB+SIFT	0.6518	0.6500	0.6373	0.6727	0.7376	0.5623
SGD+GLH	0.6101	0.6089	0.6181	0.6128	0.6742	0.5532
SGD+GLCM	0.5372	0.5375	0.5488	0.5262	0.5248	0.5502
SGD+SIFT	0.5833	0.5838	0.5981	0.5698	0.5598	0.6079
SVM+GLH	0.4896	0.4924	0.5000	0.4836	0.3586	0.6261
SVM+GLCM	0.5208	0.5247	0.5500	0.5076	0.3382	0.7112
SVM+SIFT	0.5327	0.5359	0.5617	0.5172	0.3848	0.6869
LeNet	0.6577	0.7305	0.6535	0.6535	0.6535	0.6535
AlexNet	0.6716	0.7696	0.6708	0.6711	0.6714	0.6706
AlexNet pre-trained model	0.7583	0.7941	0.8004	0.7997	0.7992	0.8009

### Lesion Classification Heat-Map

**Figure 4:**
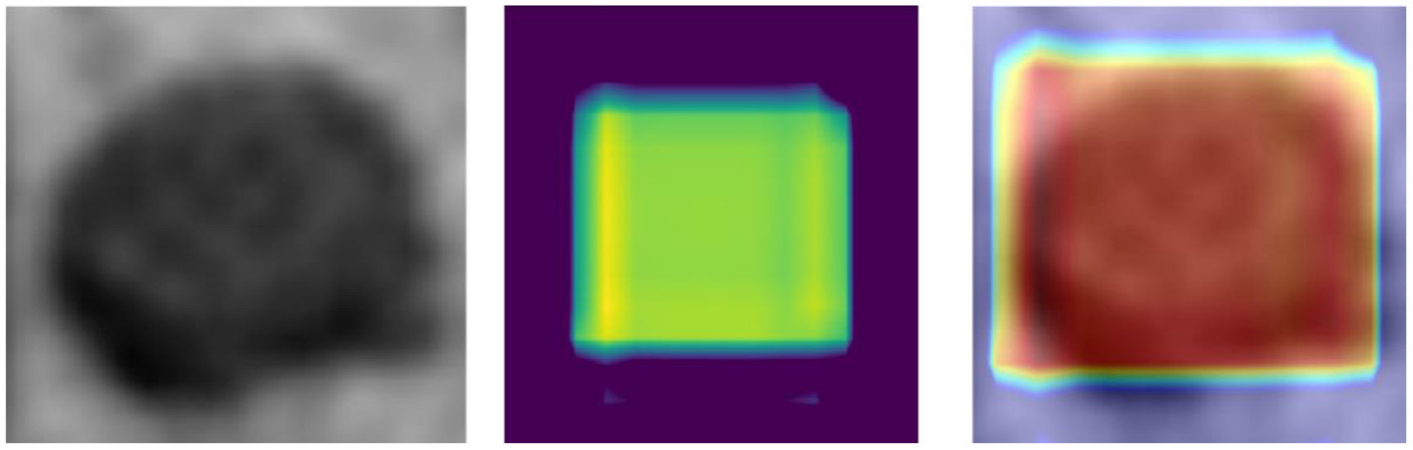
CRC LN classification heat-map (left – original image; middle – feature heat-map; right – superimposed image).

Although CNN has enabled unprecedented breakthroughs in computer vision tasks, interpretability remains unclear (Lipton, 2018). Therefore, to present a classification heat-map (Lee et al., 2019), the interpretability of the CNN model was improved. The classification heat-map identified discriminative regions (Selvaraju et al., 2017). As shown in Figure 4, the last convolution layer features heat-map was superimposed on the original MRI so that the location of the actual LN could be compared to the region highlighted by the model (Lee et al., 2019). Red regions represent class information, whereas the others correspond to class evidence.

## Discussion

First, as shown by the comparison of machine learning results, one method could yield different results if the selected features differed, and different methods would have different results even if the same features were selected. In Table 1, when each feature set was used as an input, AB, LR, and NB were better than the other machine learning methods in terms of both ACC and AUC. AB is an ensemble of DT from the view of methodology. AB utilizes multiple weak classifiers which could learn more information from the input and develop into a strong classifier when combined. Hence, AB could become a better classification method based on these weak classifiers. As a linear classification method, LR gives each input feature a weight factor which will have impact on the classification result. Based on the LR methodology, gradient descent iterates were used to find the right factors. Then, predicted values were obtained, and a sigmoid function was used for final classification. NB, with the Bayesian theorem as its foundation, is considered one of the simplest yet most powerful classification methods. A NB classifier calculates the posterior probability of input features as per the prior probability, and the input features must be independent of each other. Other machine learning methods, such as SVM and DT, performed worse. For instance, SVM is based on the kernel function which implicitly maps features into a higher-dimensional feature space and measures the distance between the feature points. Hence, the choice of the kernel function is vital. DT is based on a tree structure, which contains nodes and a directed edge. In general, there is a root node, some internal nodes and leaf nodes in a DT. The leaf nodes are decision results, and other nodes represent the features. Therefore, the higher the feature purity is, the better the classification result is.

In this study, the comparison results revealed that all other methods employed outperformed the classical machine learning methods for CRC LNM classification. LeNet and AlexNet are deep learning methods, and the AlexNet pre-trained model is a transfer learning model. In deep learning, a CNN is used to extract features from raw data layer by layer. Comparison of LeNet and AlexNet showed that the latter had better performance than the former. The reason is the structure: a deep structure performs better than a shallow one (Bottou et al., 2007; Montufar et al., 2014). The number of parameters also played a part (Tang, 2015): there are more CNN layers in AlexNet which also has more extracted features than LeNet (Lecun et al., 1998; Simonyan and Zisserman, 2014). Hence, even though the same classification function was employed, AlexNet achieves a better result. Although the problem of parameters was considered, more parameters meant more data to fit. Therefore, AlexNet needs more data, and is more likely to result in overfitting than LeNet. Overfitting hinders medical imaging analysis, and it is often difficult to collect enough medical images, like CRC LN images. In this study, the AlexNet pre-trained model solved the problem of shortage of medical data. As the AlexNet pre-trained model was trained by ImageNet, it did not need extra data for training from scratch. Hence, the pre-trained model parameters could be directly used to train new data with the help of some processing techniques, such as freezing and fine-tuning parameters. As low-level features are general, the parameters of these features could be frozen, and other parameters could be fine-tuned. In transfer learning, the learning rate is often set smaller than that for training from scratch. If the learning rate is set high, the model's parameters would be updated quickly and affect the original weight information (Wang, 2018).

As shown in Figure 4, the visualization experiment could show the model focus region of the input image. The classification heat-map represents evidence of the CNN model-based classification and could assist in clinical decision-making by directly identifying the region of interest (Lee et al., 2019).

**Figure 5:**
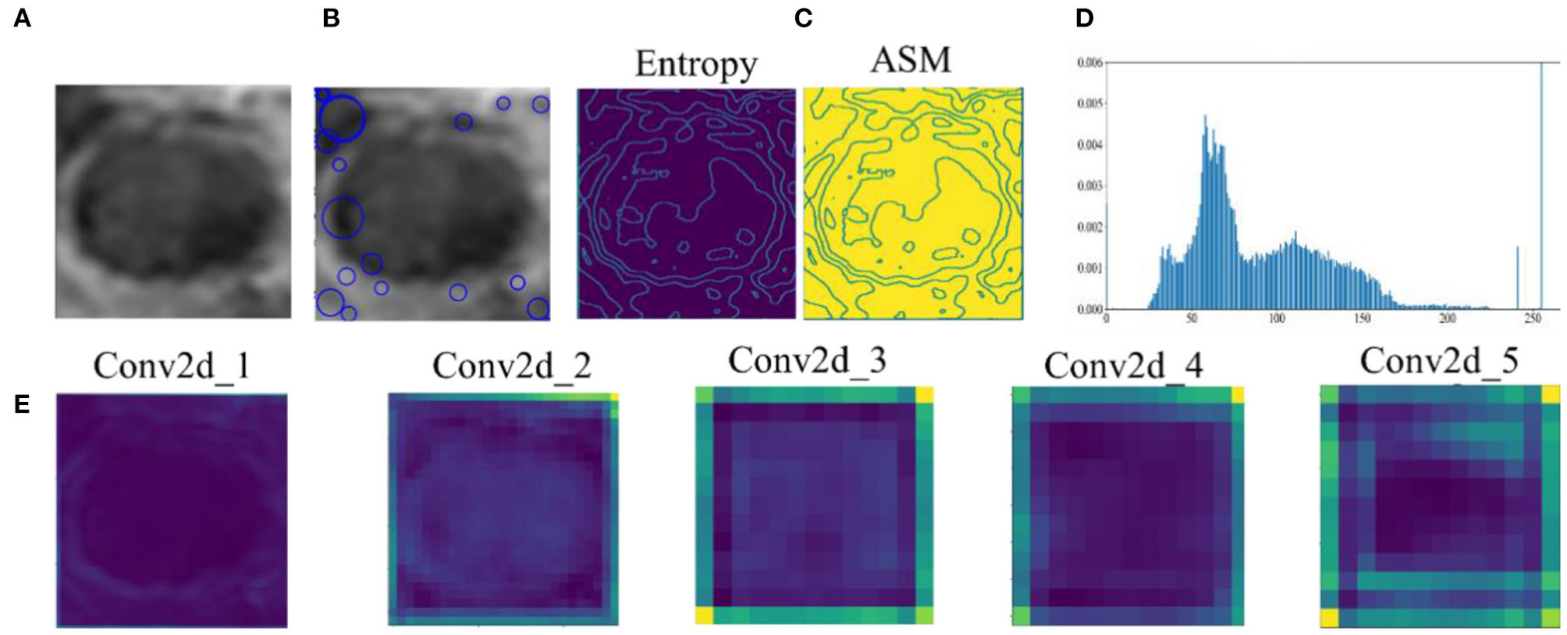
Comparison of original data and features: **(A)** original data, **(B)** SIFT feature, **(C)** GLCM feature, **(D)** GLH feature, and **(E)** features by AlexNet pre-trained model extracted from convolutional layers 1–5.

Feature extraction has direct impacts on the performance of the classification method. Figure 5 shows the original data and the features extracted by all methods. SIFT are features extracted based on the interest points of the local appearance of the original data. The more the points are, the more the features are. However, there are few points on the CRC LN lesion, whereas some points are on the edge. There is little classification information. The GLCM includes multiple-type features. Entropy and angular second moment features are listed in Figure 5. It is not easy to distinguish the region of the lesion. GLH represents the relationship between the occurrence frequency of each gray-level pixel and the gray level. Gray-level pixels could be observed. The central area of the CRC LN lesion is more than the edge area. Nevertheless, GLH does not reflect the features of the central area. The features of the AlexNet pre-trained model are from low to high levels. The features of the first two convolutional layers display the overall contour of the lesion, and others represent the semantic features of a high level. This is helpful for classification. Hence, the features extracted by the pre-trained CNN were better than those extracted by the other methods for CRC LNM classification.

Based on the experiment's results, the observations are as follows: (1) The traditional feature extraction methods are not effective in CRC LNM classification. (2) The pre-trained model of deep learning has strong transferability. Deep transfer learning applied to a small medical image dataset is better than traditional methods, and it does not need to train the model from scratch. (3) The weights of the pre-trained model realize better initialization of the parameters.

## Conclusion

In conclusion, this study showed that deep transfer learning is better than deep learning and machine learning mainly because the pre-trained CNN extracts features are more discriminative than those extracted by a CNN and artificially-extracted features. Deep transfer learning has been a popular method for image classification in recent years, and it was proved to be the best classification method among all the methods selected in this study. It could extract underlying features from raw data, does not need to select features or use raw data as input, and is less prone to user bias. A pipeline of transfer learning will be established in future studies, and the optimal deep transfer learning model for CRC LNM classification will be found.

## Data Availability Statement

The original contributions presented in the study are included in the article/supplementary materials, further inquiries can be directed to the corresponding author.

## Author Contributions

All authors listed have made a substantial, direct and intellectual contribution to the work, and approved it for publication.

## Conflict of Interest

The authors declare that the research was conducted in the absence of any commercial or financial relationships that could be construed as a potential conflict of interest.

## Footnotes

^1^Transfer learning and Fine-Tuning. Available online at: https://ru.coursera.org/lecture/machine-learning-duke/transfer-learning-and-fine-tuning-OdURo
